# 

**Published:** 1856-07

**Authors:** 


PUBLISHED MONTHLY, AT $3 PER ANNUM IN ADVANCE.

### ATLANTA
### MEDICAL AND SURGICAL
### JOURNAL.

EDITED BY

**JOSEPH P. LOGAN, M. D.,**

Professor of Physiology and General Pathology,

AND

**W. F. WESTMORELAND, M. D.,**

Professor of the Principles and Practice of Surgery.

**Pax et scientia, sed veritas sine timore.**

Vol. I. JULY, 1856. No. XI.

ATLANTA, GEORGIA:

PRINTED BY C. R. HANLEITER AND CO.

1856.

☞ Each Number contains sixty-four pages. Postage.—Under 500 miles, 15 cents a year; over 500 miles, 30

